# Reasons for low uptake of referrals to ear and hearing services for children in Malawi

**DOI:** 10.1371/journal.pone.0188703

**Published:** 2017-12-19

**Authors:** Tess Bright, Wakisa Mulwafu, Richard Thindwa, Maria Zuurmond, Sarah Polack

**Affiliations:** 1 London School of Hygiene & Tropical Medicine, London, United Kingdom; 2 Ear Nose and Throat Department, Queen Elizabeth Central Hospital, Blantyre, Malawi; Universita degli Studi di Perugia, ITALY

## Abstract

**Background:**

Early detection and appropriate intervention for children with hearing impairment is important for maximizing functioning and quality of life. The lack of ear and hearing services in low income countries is a significant challenge, however, evidence suggests that even where such services are available, and children are referred to them, uptake is low. The aim of this study was to assess uptake of and barriers to referrals to ear and hearing services for children in Thyolo District, Malawi.

**Methods:**

This was a mixed methods study. A survey was conducted with 170 caregivers of children who were referred for ear and hearing services during community-based screening camps to assess whether they had attended their referral and reasons for non-attendance. Semi-structured interviews were conducted with 23 caregivers of children who did not take up their referral to explore in-depth the reasons for non-uptake. In addition, 15 stakeholders were interviewed. Thematic analysis of the interview data was conducted and emerging trends were analysed.

**Results:**

Referral uptake was very low with only 5 out of 150 (3%) children attending. Seven main interacting themes for non-uptake of referral were identified in the semi-structured interviews: location of the hospital, lack of transport, other indirect costs of seeking care, fear and uncertainty about the referral hospital, procedural problems within the camps, awareness and understanding of hearing loss, and lack of visibility and availability of services.

**Conclusion:**

This study has highlighted a range of interacting challenges faced by families in accessing ear and hearing services in this setting. Understanding these context specific barriers to non-uptake of ear and hearing services is important for designing appropriate interventions to increase uptake.

## Introduction

Globally, an estimated 32 million children have disabling hearing impairment (HI) and the vast majority live in low and middle-income countries (LMIC).[1, 2] HI can have a substantial negative impact on language development, school performance, employment opportunities later in life, and psychosocial well-being.[3–6] There is also evidence to suggest that caregivers of children with a profound hearing impairment are at greater risk of stress, have higher out-of-pocket expenses and lose more work days than other caregivers.[3]

Early detection coupled with appropriate treatment (e.g. surgery) or rehabilitation interventions (e.g. hearing aids) are important in order to maximise functioning and quality of life for children with hearing impairments.[2, 3, 7] However, in many LMIC, there is a severe shortage of quality ear and hearing services.[8] In Malawi, there are only two Ear Nose and Throat (ENT) surgeons and three audiologists to serve a population of approximately 17.2 million.[9] Even when treatment and rehabilitation services are available, there is evidence from LMIC settings that uptake of referrals to these services can be low among children with different impairments.[10–14] However empirical evidence on the uptake of and barriers to referrals for ear and hearing services among children is lacking.

It is important to understand the barriers faced in accessing these services in order to develop tailored approaches to overcome barriers, and ultimately result in an increase in children receiving needed ear and hearing services. In this mixed-methods study, we aimed to assess the level of uptake and explore reasons for non-uptake of referrals to ear and hearing services among children in Malawi.

## Methods

This study took place in Thyolo district, in the Southern Region of Malawi between November 2015 and August 2016.

### Key Informant Method parent study

In November 2015 a study using the Key Informant Method (KIM) was undertaken to identify people with hearing impairment in Thyolo district Malawi. The KIM approach involves training Key Informants (KIs) to identify children in their communities who may have a disabling impairment and referring them to a screening camp where they are examined by relevant clinicians and referred for services accordingly.[15]

In the study in Malawi, 29 community health workers (known as Health Surveillance Assistants, HSAs) from five randomly selected health centres in Thyolo district were trained by an ENT surgeon to be KIs. The HSAs were trained in Primary Ear and Hearing Care (PEHC) using the World Health Organization (WHO) Basic and Intermediate training modules on PEHC.[16] The training had theoretical and practical components. The practical component included: history taking, ear and otoscopic examination, and voice testing. Following the training, HSAs were asked to identify adults (>18 years) and children (<18 years) with ear conditions and/or hearing loss in their communities and invite them to attend a screening camp at a selected health centre. HSAs used multiple methods for identification, using the skills learnt in the training. This included door-to-door visits, and school screenings.

Screening camps were conducted by an ENT Surgeon, ENT Clinical Officers and Audiology Officers from Queen Elizabeth Central Hospital (QECH). Children underwent a hearing test hearing (using otacousitic emissions tests for <4 years and Pure Tone Audiometry for 4+ years) and examination of the ear using otoscopy. Participants were referred to ear and hearing services at the QECH, as appropriate. For example, children with chronic suppurative otitis media were treated with ciprofloxacin ear drops and referred to QECH for surgery and children with suspected sensorineural hearing loss were referred for further audiological assessment and possible fitting of hearing aids. The QECH, in Blantyre, is the largest hospital in Malawi, and one of the few central hospitals with ENT and audiology departments. Services at QECH are free at the point of care. In total, HSAs identified 1739 people (adults and children) with suspected ear disorders or hearing loss. Of these 860 attended camps, 484 of whom were children, who are the focus of this paper. Of these children, 170 were referred to QECH for ear and hearing services.

### Quantitative survey

A follow-up survey to the KIM study was conducted in June 2016 to assess the uptake of referrals. All households of the 170 children referred to QECH were invited to participate. The primary caregivers were interviewed using a structured pre-coded questionnaire, in private, at a central location in the village (e.g. a health post or school) (S3 File). Interviewees were asked whether they had attended the referral(s) and if not, the reasons why. Reason for non-uptake was asked as a single open question with pre-coded response options developed based on previous research, discussions with stakeholders and pilot testing.

### Qualitative study

Informed by the quantitative study, a qualitative study was undertaken to understand the barriers to referral uptake in more depth. Semi-structured interviews were conducted with caregivers of children identified in the KIM study who did not take up their referral to QECH as well as stakeholders.

#### Study sample

Purposive sampling was used to select a sub-sample of 30 children (<18 years) who did not take up their referral. The sample was selected to ensure representation from different health centres, child age, sex, and severity of hearing loss. Interviews were conducted with the main caregiver.

#### Data collection

Interviews with caregivers were conducted at the local health centres and lasted approximately one hour. Topic guides were developed that included a range of open-ended questions. These were piloted and revised during the data collection period in light of the emerging themes. Caregiver interviews covered: history and impact of the child’s ear and hearing issues, experiences at screening camps, and barriers faced in attending the referral (S1 File). For stakeholders the interviews explored their perspective on the barriers experienced by families at the family, community, screening camp, and hospital levels and recommendations how to address these (S2 File). Interviews were audio-recorded and detailed field notes taken. The recordings were transcribed and translated.

#### Research team and reflexivity

Two experienced researchers conducted the interviews: a male Malawian researcher (RT) together with a female UK-based researcher (TB). For stakeholders who were proficient in the English language, the interviews were conducted by TB in English, and the remaining were conducted in Chichewa by RT.

#### Analysis and findings

Transcripts were coded by two researchers, and data was managed by nVivo (Version 11). A thematic analysis was used; data was coded into key themes and sub-themes through an iterative process, and a constant comparison of emerging issues identified between the two researchers.[17]

## Ethics

Informed written or thumb-printed consent was obtained from all study participants. Ethical approval was granted by the College of Medicine Research and Ethics committee in Malawi and the London School of Hygiene & Tropical Medicine ethics committee.

## Results

### Quantitative survey

Out of the 170 families of children who were referred for ear and hearing services, 150 were traced (88% response rate). All caregivers interviewed were female. The majority of the children referred were female (74%). The children ranged in age from 0–18 years with a mean age of 9.4 years (95%CI 8.7–10.2).

Only five out of the 150 children (3%) had attended their referral at QECH. The remaining 145 were interviewed about reasons for non-uptake of referral. The most commonly reported reasons for not attending referral services were transportation difficulties (41%), lack of information or knowledge about the referral process (60%) and financial barriers (33%) (Table 1). The main reported financial barriers included lack of money for transport (28%), and food (21%). In addition, 40% of caregivers reported that they expected someone, such as a community health worker, to visit the family to follow-up with them.

**Table 1:** Reported reasons for not attending referral services (n = 145).

	Number reporting	%
**Transport difficulties (practical/geographic challenges not including cost)**		
No Transport available	35	24%
Distance too far	36	25%
Unable to carry child	2	1%
Not safe	1	1%
Total*	**59**	**41%**
**Lack of information/understanding**		
Not enough information about referral	73	50%
Location referral wasn’t specified	22	15%
Unclear if service would cost money	4	3%
Total*	**87**	**60%**
**Financial**		
Not enough money for transport	41	28%
Not enough money for the service	6	4%
Not enough money for food needed	31	21%
Total*	**48**	**33%**
Told health worker would visit family but did not happen	58	40%
Forgot appointment	3	2%
Afraid	13	9%

*More than one response permitted explaining why category sub-totals are less than sum of individual responses

### Qualitative follow-up

In total 23 caregivers were interviewed. For seven selected child/caregiver pairs, the HSAs were unable to locate the families. We did not select additional families for interview, because theoretical saturation was reached (i.e. no new information was emerging from the interviews). Table 2 shows the characteristics of the children included in the sample. Of the children of school going age, the majority (83%) attended school. However, 93% of these children were in a lower than age appropriate grade.

**Table 2:** Characteristics of children in qualitative study (n = 23).

	N	%
**Age group**		
0–4 years	4	17
5–10 years	9	39
11 years+	10	43
**Sex**		
Male	10	43
Female	13	57
**Diagnosis**		
Normal hearing with ear disorders	5	22
Mild hearing loss	3	13
Moderate hearing loss	5	22
Severe hearing loss	3	13
Profound/probable profound hearing loss	4	17
Fail OAE (one or both ears)	3	13
**Referral**		
Surgery	9	39
Hearing aids	7	30
Unknown	7	30
**School attendance**		
Yes	15	83*
No	3	17*
N/A (<6 years)	5	-
**Repeated grade**^		
Yes	14	93
No	1	7

* % of those eligible for school (n = 18)

^ % of those attending school (n = 15)

In addition, we interviewed 15 key stakeholders within Thyolo and Blantyre districts involved in ear and hearing care and the screening camps. At least one key stakeholder from each health centre was interviewed as well as staff at the district hospital in Thyolo and QECH. A table of the key stakeholders is provided below (Table 3).

**Table 3:** Key stakeholders interviewed.

Stakeholder role	Number
Health Surveillance Assistant supervisor	1
Health Surveillance Assistant	4
Medical Assistant	5
Ear Nose & Throat clinical officer	2
Audiologist	1
Malawi Council for the Handicapped (MACOHA) staff member	1
Chief clinical officer	1
Total	15

Seven key themes described non-uptake of referral by stakeholders and caregivers.

#### 1. Location of hospital

The distance to QECH was perceived by most caregivers to be vast and a significant obstacle to taking up the referral. For example, one caregiver explained that their village was 100km from Blantyre and, because of the challenging terrain, the journey would be at least 2.5 hours. It also required walking or cycling up steep hills to reach public transportation. Thus the journey was perceived to be challenging particularly for their children. Another caregiver described the challenges of making this journey particularly in the context of concerns that they would not be seen on the same day at the clinic:

*It’s a long journey*, *imagine from here to Goliati you will ride a bike and in the hills you will be walking on foot*. *At Goliati we board another [minibus] to Limbe and then another to Queens*. *Its long journey and you might not be assisted the same day when you go*. [Caregiver 14]

#### 2. Lack of transport

Several caregivers and stakeholders reported that transportation services, both public buses and ambulances, were not easily available to travel to Blantyre. Some explained that ambulance services at the health centre and district hospital level, in theory, can transport patients to the next level referral facility (i.e. from the health centre to district and district to QECH). However, this service was reported to be unreliable or only used for priority services such as maternity care. One caregiver explains their unsuccessful attempts to use the ambulance service:

*We tried that time to get an ambulance but failed because every time we came to ask about the ambulance*, *we were told that it had already left*. [Caregiver 14]

Further, stakeholders reported that the ambulance service to QECH was one-way only and once patients were in Blantyre they faced the additional challenge of finding a way back to their villages.

*The other challenge is when they are discharged*, *because coming here is easy because they have an ambulance*. *When everything has been done here and they have been helped or they have been assisted*, *they still need an ambulance to take them back*, *so we don’t have a ready ambulance to pick them up*. [Stakeholder 9]

#### 3. Indirect costs

Although most health care in Malawi is free at the point of delivery, over a third of caregivers raised concerns about indirect costs of seeking care associated with travel and time spent at QECH. Some reported that, because of the long distance, from their village to QECH it would cost around 1500 Kwacha, a price which is prohibitive for rural farming families.

A few caregivers mentioned that if they requested an ambulance, they were told to buy fuel for the journey, which they could not afford. In addition, there were also difficulties with paying for food required for both the journeys and time spent at the hospital.

*Some things might be needed*, *[to travel to and wait at QECH] such as flour*, *firewood and relish and some other things like porridge flour*, *sugar and others*. [Caregiver 1]

Some caregivers explained that their income depends on seasonal activities; at certain times of year they are not engaged in income generating activities making it difficult for them to meet the additional costs involved in care seeking. Many also felt concerned that seeking care would be a lengthy process resulting in several days where they could not be engaged in work on their farms. Further, some of the caregivers reported that there would be nobody to care for their other children if they travelled to QECH for several days. These interacting challenges were summarised by one caregiver:

*From here [Chisoka] to Queens there is a need for proper transportation as you know this place is far*. *And when you arrive there you know these days [outside harvesting season]*, *people don’t harvest enough and it is possible that we might not be treated same day*, *maybe we may spend some days*. *So [money for] food would and transport would be a problem*. [Caregiver 9]

#### 4. Fear and uncertainty of the hospital

Several factors relating to the referral hospital were mentioned by caregivers and stakeholders as potential barriers to the referral uptake. Many caregivers and stakeholders expressed fear and unfamiliarity of QECH as a reason for not attending. Most caregivers had never previously been to QECH, which was perceived as a “big hospital”. Several caregivers asserted a fear that they “would not know where to go” once they arrived at the hospital. Further, fear of long waiting time at the hospital was raised as a concern by some caregivers.

*Some people think that Queens is a very big hospital*, *you can spend the whole day without being helped*. [Caregiver 7]

*It is just fear*, *some have never been to Queens so referring them to Queens… and you give them directions*. *They may have money but for them to go*, *maybe it is fear*. [Stakeholder 6]

#### 5. Procedural challenges in the camps

The interviews revealed a number of issues with communication at the screening camps that negatively influenced uptake of referral. As part of the camp protocol, caregivers were verbally advised to attend QECH and this was noted in their health passport. HSAs were instructed to then follow up with patients to check attendance to QECH and ensure they adhere to treatment or instructions. However, it was evident that many caregivers were confused about the referral process. Many caregivers reported that either they were not aware that they had been given a referral at all or that they were waiting to receive more information about when to attend QECH. Where possible, we examined health passports for the referral and found referral notes were lacking on several occasions. Caregivers explain the lack of information:

*I was not told that we needed to go*. *We were just waiting for information on the day to go to Queens*. [Caregiver 1]

Several caregivers expressed a motivation to take up the referral. However, lack of information about the referral in the camps together with other barriers prevented them from taking it up. One caregiver explains this:

I: *But if you were told to go to Queens would you have managed to go*?

P: *Yes*, *I would have gone… perhaps transport would have been difficult*. *I would first have looked for transport and once found then I would go* [Caregiver 5]

This caregiver also mentions difficulty with transport, highlighting the multitude of barriers faced in this context. Screening camps were reported to be very busy and the majority of caregivers described long waiting times to be seen by the clinicians. Most caregivers reported that the results of the tests were not explained by the doctor in the camps. As a result, caregivers expressed an uncertainty about what would happen at QECH. Some caregivers mentioned that they were just told that the problem was “big”. In addition, caregivers were not given information on how to manage their children while waiting to go for referral. Stakeholders also highlighted the lack of information given to caregivers as a substantial shortcoming of the camps, as explained by one stakeholder:

*Of course giving them advice*, *advising them on what to do with the conditions*, *because they needed counselling for them to understand the problem*, *if the problem could be treated*, *or how can they be assisted with their problem*. *We didn’t have that time*, *and we just said no*, *just go to Queens and you will be treated or come to Thyolo you will be treated in such a way*. [Stakeholder 8]

#### 6. Awareness and understanding of hearing loss

The majority of stakeholders felt that limited knowledge of ear and hearing health for most people living in the rural areas of Malawi was a substantial barrier to uptake of referrals, and some also indicated that children with disabilities may be neglected by the their families.

*The most important issue which is like a barrier for them to access the services it is; themselves*, *because sometimes they don’t even know*, *even understand what is going on*, *so at the end of the day they don’t give them [the child] a second chance*. *They just declare that this is the way things are*. *Maybe you’ve heard somewhere that these kinds of children*, *or the disabled*, *people would just dump their house and just sit there*. [Stakeholder 11]

Interviews with caregivers suggested that specific knowledge regarding the causes and available treatments for their child’s ear and hearing loss was limited, despite attendance at the KIM screening camps. Some caregivers also described seeking alternative or home-based treatments for their child. For instance, distilling cooking oil or traditional medicines in to the ear canal. However, many acknowledged that no improvements were seen post-treatment. Despite this, many of the caregivers did display an awareness of their child’s hearing loss. Most were able to recall when their child’s hearing loss or ear condition started, even if it was delayed and several described the impact on their child. For example:

*We can say that the problem started at birth but then for us to realise her difficulty in hearing was when she was 4 years old*. *That’s when we realised that the child does not hear properly because when spoken at if she was not looking at you then she was acting in way like she hasn’t heard you while if she is looking at you*, *she was able to hear*. [Caregiver 5]

In contrast to the stakeholder perceptions, caregivers did appear to be motivated to seek care for their child. They attended KIM camps and the majority of caregivers (n = 18) interviewed had also previously sought treatment at health centres for their child’s ear or hearing problem.

#### 7. Lack of availability and visibility of ear and hearing services

As well as the specific challenges to uptake of referral to QECH, the interviews raised more broadly the lack of resources at health facilities as a serious problem limiting access to ear and hearing services. Several stakeholders highlighted the lack of visibility of the ear and hearing services at QECH and Thyolo district hospital as a barrier to patients receiving appropriate care. They felt that because other staff at the hospitals were not always aware of the ear and hearing services, patients do not always actually reach the ENT department. Instead patients may be sent from department to department without ever finding the appropriate provider.

*What is working well is; at least there is somebody who deals with these issues like the ENT clinician*, *where it doesn’t work well is; these other people who are not ENT clinicians*, *they don’t know what to do and they may send back some of the children when they are not supposed to be sent back*. [Stakeholder 11]

Some stakeholders felt that these experiences would make people reluctant to seek care again. This was supported by caregivers who said that if they attended QECH, there was a risk of not receiving assistance at the hospital on the same day. This perceived risk discourages them from spending the money to get there:

*We might go there and may not find the doctor*. *We only have money for one day [so] we may be stranded*. [Caregiver 14]

Limited availability of the ENT personnel, in general, at health facilities was raised as an issue by both caregivers and stakeholders. For example, some caregivers reported previously attending the district hospital, which often involved a day of travel, but finding that the ENT clinical officer was not available and therefore no care was received. Stakeholders attributed this to lack of ENT personnel.

*They think of transport issues*, *and how they will reach there if they will be admitted or how they will meet the ENT person since its only one person*. *Sometimes he is not there*, *he goes to the meetings*, *and there is no one to help them on the issues of hearing problems*. [Stakeholder 1]

Several caregivers also asserted that health facilities were not able to provide treatment for ear problems because they did not have drugs and once they had experienced this, they did not feel it was worthwhile to seek ear and hearing services again. The lack of adequate medication, equipment and human resources to enable diagnosis and appropriate treatment for children with ear and hearing problems was raised by several stakeholders. For example, health centre staff described the challenges of managing ear conditions due to limited resources and expertise:

*We don’t look into the ear*, *we just see if the child is discharging*, *we look at how the pus looks like and give them a cotton to wipe with but we don’t look inside because we don’t have the equipment to use*. [Stakeholder 4]

## Discussion

Uptake of referrals for children with ear and hearing issues was extremely low (3%) in this study setting. In the survey, transport difficulties, lack of information regarding the referral and financial constraints were most commonly reported as reasons for non-uptake. The semi-structured interviews enabled us to explore these barriers in more detail and highlighted that, while caregivers appeared to be motivated to seek care for their child, several often-interacting factors prevented them from doing so. These included location of/distance to the hospital, indirect costs, lack of transportation, procedural challenges in camps, awareness and understanding of ear and hearing issues, fear and uncertainty about the referral hospital, and lack of availability/visibility of hearing health services.

Delayed or of lack of access to appropriate health and rehabilitation services can have substantial long-term consequences for children and their families, including poorer health and quality of life, increased risk of mortality, lower rates of school participation and a greater risk of poverty.[18] To avoid these consequences, efforts to tackle the identified barriers are essential. The findings of this study suggest a multi-dimensional approach may be required to increase uptake of referral for ear and hearing services.

Several barriers raised in this study (distance to hospital, lack of transport, and indirect costs) concur with previous literature on challenges in accessing health care services in poor, rural settings.[18, 19] For example, in the 2015–2016 Malawi Demographic Health Survey, distance to health facilities was the most commonly reported problem by women in accessing care for serious health conditions (56%).[20] Despite free health services at the point of care in Malawi, many families in this study were unable to afford the in-direct costs of seeking care such as transportation and food. This aligns with a previous study in Malawi, which found that that economic hardship and distance to health facilities decreased acceptance of free cataract surgery for children highlighting the often prohibitive indirect costs of seeking care.[21]

Additional specific challenges were raised in this study such as procedural challenges in the screening camps resulting in a lack of information about the referral process. This concurs with findings from a similar study in Bangladesh following KIM screening camps for children with disabilities, where confusion and misunderstanding about the referral process contributed to non-uptake of referral.[13] The interaction between health workers and services users is well recognized to be an important factor in uptake of services, although this has not been well explored for children in low-income settings.[22, 23] In the current study caregivers were verbally informed about the referral, but the interviews revealed that they were still uncertain about the referral process. Screening camps were reported to be very busy, which may have limited the time specialists spent explaining the referral as well as making it more challenging for caregivers to absorb the information. The lack of information also likely contributed to caregiver’s fear and uncertainty about attending QECH. Fear of surgery has been highlighted in previous studies in LMIC as influencing lack of uptake of referrals for example for clubfoot and cataract procedures.[24–26]

These barriers highlight a critical need for more effective communication with caregivers and children in future KIM studies and other community outreach activities that involve onward referral to health services. A core component of these activities should be dedicated personnel who appropriately communicate information on diagnoses and recommended action and create a safe space for caregivers and children to discuss their concerns and questions. In this setting, this could be delivered, for example, through the HSAs, other dedicated trained counselors or peer educators (e.g. people from same community who have previously used ear and hearing services). There is evidence that health education interventions (such as structured group education, or use of pictorial cards) delivered by health care workers or community health workers can have positive effects on uptake of health interventions for children including treatment commencement for malaria in Nigeria, and child vaccination uptake in Pakistan.[27] Further research is needed to assess the effect of educational or counseling interventions on uptake of ear and hearing referrals in the Malawian context.

The limited availability of human resources for ear and hearing care was highlighted in this study. This a significant challenge throughout the African region–with many countries having less than 1 ENT per million population.[28] In this context, delivery of services at the community, to tackle geographic and financial barriers, could be achieved through training of community health workers in basic ear care alongside outreach activities conducted by ENT specialists. The effect of delivering of services close to home has been evaluated in several African countries with promising results.[27] [29] The WHO Programme for Prevention of Deafness and Blindness advocates for a “task shifting” approach to managing ear and hearing conditions in the community and provides training materials in primary ear care.[30] In this Malawian setting, one strategy could be to train HSAs to identify and manage basic ear and hearing conditions in the community. Training health-centre staff in the management of simple ear issues, such as the removal of impacted wax, also warrants investigation. However, evidence is required on the feasibility and effectiveness of the WHO training materials and the task shifting approach, in light of competing time demands for health workers. Key lessons about integrating basic ear care into primary health services may be drawn from the field of blindness, as primary eye care has been introduced in many countries in sub-Saharan Africa, including Malawi and Rwanda.[31]

Although “task shifting” to non-specialist staff can relieve the burden on secondary and tertiary services, certain interventions such as surgery cannot always be readily provided in a community setting. Thus the journey to QECH may not always be avoidable. This research highlighted that ambulance services are available from the health centre to QECH, however certain health conditions (e.g. maternal health) are prioritized. This is not surprising in a resource constrained setting such as Malawi. However, with the growing burden of non-communicable diseases in LMIC, ambulance services provision for non-emergency health conditions should be considered.[32] We also found that ambulance services are not always available for the return journey to Thyolo, resulting in families becoming stranded at QECH. Careful planning of return services to Thyolo from QECH warrants further attention.

Other interventions that deserve attention include the use of text messages to remind patients about appointments and provide health education which has been found to be effective for increasing uptake of health services.[27] Further, awareness of ear and hearing service availability at QECH must be raised amongst general medical staff at the hospital to avoid caregivers being turned away, and assist navigation to ENT department. This could be approached through sensitization meetings and display of information across all departments.

### Strengths and limitations

This study had several strengths. We used a mixed-methods approach to quantify uptake and key barriers to referral uptake, and then explore these barriers in more depth. We interviewed caregivers and stakeholders in order to explore different perspectives. In doing this we were able to triangulate the barriers reported by the different groups of participants.

There were some limitations, which need to be taken in to account. Qualitative interviews were conducted at health centres for pragmatic reasons and caregivers may have responded differently compared to if they were in their home. For example, they may have felt reluctant to fully express concerns related to the camps that were held at the health centres. Efforts were made to limit this risk by ensuring the interview room was always private, health centre staff were not present and experienced researchers familiar with qualitative interview techniques conducted the interviewers. We purposively sampled the children according to degree of hearing impairment and/or ear disease as well as age. This was done to reflect the types of children who were referred to QECH. It is possible that children with more severe impairments experience different barriers that could not be explored in depth within this study due to limited numbers included in the study.

## Conclusions

Very few children identified in the community as needing ear and hearing services attended their referral appointment. Families referred to QECH for ear and hearing services experienced a range of interacting barriers which contributed to non-uptake. Understanding these context specific barriers to non-uptake of ear and hearing services is important for planning services and designing interventions to increase uptake.

## Supporting information

S1 File Topic guides for caregivers. (DOCX)

S2 File Topic guides for stakeholders. (DOCX)

S3 File Data collection form for quantitative survey. (DOCX)
