# Supplementary material for: Reasons for low uptake of referrals to ear and hearing services for children in Malawi
Source: PLoS One. 2017 Dec 19;12(12):e0188703. doi: 10.1371/journal.pone.0188703 (PMC5736203; doi:10.1371/journal.pone.0188703)
Supplement: S1 File — (DOCX) [file pone.0188703.s001.docx]

**Barriers to the uptake of ear and hearing service referrals for children in Malawi**

**Semi- structured interview guide** – with Primary Caregiver

**Introduction:**

Good morning and thank you for your time. I am ___________ from .......... I am here today to as part of the research study we discussed before and which you kindly agreed to take part in.

Remind parents of the full information sheet that they received about the study. Remind them of the issue of confidentiality which is fully explained in the info sheet.

You can stop me at any time if something is unclear. If there us anything that you do not want to answer, then you do not need to.

| **Code:** |  |
| --- | --- |
| **Interview Date and Time:** |  |
| **Interview Venue and Location:** |  |
| **Interviewer:** |  |
| **Interviewee:** |  |
| **Relationship to Child:** |  |
| **Gender:** | - **Male** - **Female** |
| **Child in School:** | - **YES** - **NO** |
| **Type of School:** | - **Primary** - **Lower Secondary** - **Upper Secondary** - **Special School** |
| **General Observations:  (Interview location, who present, anything which might impact how the interview is conducted, e.g. other present.)** |  |

# About your Family (Icebreaker)

Please tell me about your family

1. Prompts: Who lives in the house? Number of children living in the household? (age, sex) Are all the school-age children going to school? If not, why not? **What grade are they in at school?** **Is this age appropriate grade?** Who is working in the house? What kind of work?
2. Who is involved in caring for the child?

# About the child’s ear or hearing issues

# Understanding of hearing loss and causes

# Please tell me about your son/daughter who was seen at the camps in (Jan)

- *Get a sense of understanding of child’s hearing difficulties or troubles with ears (check diagnosis, if conductive/chronic middle ear issues questions might be slightly different)*
  - How is [name’s] hearing?
  - When did you first notice it? Did you seek care at the time? Why/why not? Who first noticed the issue? Was it noticed at home or at school?
    - *If problems with middle ear:* How many times has he/she had this issue? When was the first time? What usually happens when he/she has the issue?
  - Diagnosed prior to the camps?
    - - If yes, when?
      - Where?
      - Who went with the child?
      - What happened?
      - What were you told?
      - Did the child receive any treatment?
        - If so, what was that treatment?
      - Did it make a difference for the child?
      - If the treatment stopped or child stopped using the device, why was this?
      - If treatment was surgery or medication, explore if the issue recurred (eg. recurring ear infections, perforations, discharge). Any complications from the surgery?
      - If problem has happened more than once, how often did it happen? did you seek care each time?
  - What do you think caused the condition?
  - Why do you think the child has the condition?
  - What do your family/husband think?
  - Do you know anybody else with this condition? Other family members? Did you know about the condition before your child experienced it?
    - Note: some children in sample have family history, might be worth checking if anyone else in the family has any problems with their hearing and how it affected them.
    - Did they seek care for the sibling? Or other family member?
- Has the child ever seen a traditional healer because of the ear/hearing problem?
  1. Impacts of hearing loss
- Does hearing loss have an impact on [name’s] life? (*Note: If no, move on. If yes, explore what that impact might look like.)*
  - Pick up on any issues noted. Eg. unable to speak, explore how that affects family/child.
- What things does she/he find more difficult or not possible for your child to do, that other children of the same age can do? Please tell me about his/her day – does she go to school/is able to help around the house (kind of assistance needed)
- If child goes to school, how is he/she going? Any difficulties? Any support from the teacher
- What is she/he able to do?
- How does it impact parent’s life/other family members?
- How do other family members treat your child?
- **Communication difficulties?**
- **How do you try to overcome the communication difficulties?**
  1. Interviewer note: any other observable health conditions hearing loss?
- We have talked a lot about [name’s] hearing, are there any other concerns you have about their health? (*Separate this question from hearing unless it comes up naturally)*
- If yes, ask about care seeking for that condition? Eg. Do you have to go to the health centre for this and how often?

# Treatment-seeking behaviour – understanding of diagnosis and referral

(Interviewer Note: This is NOT about checking up on them for not going – emphasise about learning from their experience)

### *Check what they recall about the screening camp and understanding of diagnosis/referral:*

1. Do your remember the ‘camp’ – held at XX (camp location) - which you attended with your child in Jan/Feb this year. We would like to learn more about your experience from this camp.

- How did you hear about the camp?
- What did you think might happen at the camp?
- What happened at the camp? How long did you wait? **How far did you travel? How did you travel?**
- What were you told about your child’s ear/hearing? **Did the doctor explain the results to you?**
- What were you told about possible services available to help your child?
- Did you understand what the doctor told you?
- (*Note: if required hearing aid:* Did they tell you that you needed a small gadget to help your child hear? Do you know what a hearing aid is? *Show picture of a child wearing hearing aid.* Do you know how much this hearing aid might cost?
- Note: If surgery required: did caregiver understand what that surgery would involve? How much it would cost?)
- Do you remember being given any referral form, notes written in health passport or advice?
- Can you show me what you received?
  - If yes – what did you do?
- What did you understand would happen at the follow-up appointment?
- Do you think your child’s ear/hearing problem can be improved (or cured). If yes, how?
- Note: if they say they were waiting for information: did the health surveillance assistant talk to you about going to Queens?

1. Past experiences

- Also ask about past experiences of services that may inform their decision to attend/not attend a new referral (positive/negative experiences). What kind of treatment do you like?
  - When someone is ill, what do you do?
  - Something here about serious illnesses, would they go to QECH? Priorities?
  - Where is the nearest clinic?

1. **Barriers to attending referral**

(Explore main reasons for not attending referrals - aim to get concrete examples where possible rather than generalisations)

- What did you understand about the referral? *(Note: if identified difficulties, pick up on these)* What was the referral?
- What are the main reasons are for your child not going to the hospital for their hearing problem? What might be some of the challenges you or your child face in accessing help for hearing? **Prompt more, ask specific questions about each barrier that is arising.**
  - *Note: Prompt if necessary: eg. a lot of caregivers have said that [cost, knowledge of cost, transport, availability of information on service, stigma, lack of time, family members] was the reason why they did not go to hospital. Explore these topics further. Eg. If transport is a problem, why? What are the distances – how long to get there, cost of transport, does someone have to accompany the mother and child, time off work etc, caregivers for other childen? If cost is perceived as an issue: how much do services cost? Is it difficult for you to pay for services?*
  - *If there are a number of reasons, ask parents to rank the main reasons and explain why they have ranked them in that way (A small ranking exercise here on card)*
- What do you think would happen at the hospital if you attended with your child? **Were multiple followups required?**
- Did you know what was going to be offered when you attended the appointment at the hospital?
- Would you like to followup on your referral? If no, why not?

1. **Enablers to attending hospital**

- What might be useful for overcoming some of the challenges that you mentioned? Are there any suggestions you have for improving uptake of services? Is there anything that you think would help your child to attend their hospital referral. What support would you like to attend the referral?
- If the parent was told the child needed a hearing aid, do they think that is worth investing in?

**SUMMARISE MAIN POINTS OF INTERVIEW BACK TO PARTICIPANT, TO CHECK UNDERSTANDING. Is there anything else that we haven’t covered about that you would like to add? Is there anything else you would like to tell me about your situation?**

Thank you for your time. We will be providing feedback through the XXX. This will be in 2-3 months time , once we’ve had time to look at all the information from the parents and children.
