# Supplementary material for: Reasons for low uptake of referrals to ear and hearing services for children in Malawi
Source: PLoS One. 2017 Dec 19;12(12):e0188703. doi: 10.1371/journal.pone.0188703 (PMC5736203; doi:10.1371/journal.pone.0188703)
Supplement: S2 File — (DOCX) [file pone.0188703.s002.docx]

Stakeholder topic guide

1. Can you tell me about your role?

**Prompts:**

- How long have they been in that role,
- What is their main responsibility
- How many staff are they responsible for?
- What geographic area they cover (national or regional/local remit).
- How does your work relate to children with hearing loss
- What are the common issues you see at the health clinic with ears and hearing? What is the main reason parents usually bring their child?
- How are people referred to you?
- How old are the children that normally come?
- What is done to manage ear and hearing issues at this level? What services are available for people with hearing loss?
- How are you connected with QECH?

So you have been in your role for XXX years, and have a wealth of experience from which we can learn from. It will be great to hear more about this.

We would like to explore the main facilitators and barriers to the uptake of ear and hearing services for children in this setting. Let us start with what you think might be helping people come for services starting with at the family level.

# At a family and community level

- What do you think the main challenges which families face in accessing health services generally and specifically for children with hearing impairments (prompts: transport, finances, attitudes, perceptions of priority, lack of knowledge, past experiences of health services)?
- What do you think are the enablers for attending services? (currently)
- What do you think are the main attitudes towards children with hearing impairments and their access to health services?
- Are there any cultural beliefs about hearing impairment?
- What are the attitudes towards children with hearing impairments and their access to health services?
- Are children with hearing impairment included in education, other services? Excluded? Other health services?

# At screening camp (provide description for those who were not involved?)

- What do you think are the benefits/shortcomings of using this approach for referring children with hearing impairment?
- What improvements do you think could be made?
- How do you think families generally understand the need for services for their child after referrals are made at the camps?
- For HSAs
  - How did you go about identifying children in the community for the camps? What did you usually say when you were informing them about the camps?
  - Were you aware of how the referral process would work? Or How did you understand the referral process would work?

**At hospital**

- Once children/families arrive at the hospital, what do you think are the barriers in place at this level?
- Do you think there are any issues which affect inclusion of children in health and rehabilitation services (prompts: accessibility, communication difficulties, waiting times, staff attitudes)

# **At a national policy level**

- Get an overall picture of who is responsible for treatment and rehabilitation provision for children with hearing impairment at the district level? Which ministerial offices responsible for what? How is government policy impacting upon access to these services? What’s working well, less well, change as a result of any new policies.
- How does your role fit in to the national picture? How do you connect with other health workers in the system- at other levels?
- Prompts: disability legislation, policy Issues related to the policy environment, implementation of the policy, budgetary allocation, ministerial roles and responsibilities for children with disabilities, training of health staff

# Recommendations

- Recommendations to address some of the issues above (refer back to specific issues) to improve uptake of ear and hearing services for children with hearing impairment
- What improvements do you suggest in your health centre? At the family level? At the national level? At the hospital level? At the screening camps
- Eg. You mentioned that there were problems with XXXX, how do you think this could be overcome?

# Other

- Are there any other important issues which I haven’t covered which you would like to comment on that will be useful for us to address in relation to uptake of ear and hearing services for children?

Thank you for your time. We will be providing feedback through the XXX. This will be in 2-3 months time , once we’ve had time to look at all the information from the parents and children.
