# Supplementary material for: Reasons for low uptake of referrals to ear and hearing services for children in Malawi
Source: PLoS One. 2017 Dec 19;12(12):e0188703. doi: 10.1371/journal.pone.0188703 (PMC5736203; doi:10.1371/journal.pone.0188703)
Supplement: S3 File — (DOCX) [file pone.0188703.s003.docx]

**Malawi KIM Questionnaire**

**Kalata ya mafunso ya ku Malawi**

ID number:

Traditional Area (please specify): *mfumu: (Tchulani chonde)*

Home village (please specify): *Mudzi ochokera (Tchulani chonde)*

Age (please specify): *zaka zobadwa (chonde Thulani)*

Gender: *Wamkazi kapena Wamuna*

☐ Male *Wamuna*

☐ Female *Wamkazi*

If your child did not attend any services, why not? (check all that apply):

*Ngati mwana wanu sanalandire thandizo, mukuganiza kuti ndi chifukwa chiyani?*

☐ Transport difficulties *vuto la mayendedwe*

☐ Did not have enough information about the referral *sindinamvepo*

☐ A location was not specified *malo opitawo sindikuwadziwa*

☐ Was told someone would call back at a later date, which did not happen

*anangoti abwera sanabwerenso*

☐ Did not see improvements, *panalibe kusintha*

☐ Child not well *mwana sanali bwino*

☐ Other family members *unwell matenda kunyumba kwathu*

☐ Unclear if the service would cost money *ndimaona ngati ndi zolipilitsa*

☐ Not enough money for transport *ndalama ya transport panalibe*

☐ Not enough money for the service *sinkanakwanitsa chifukwa chosowa ndalama*

☐ Not enough money for the food needed on the journey *ndalama panalibe zodyera poyenda ulendo.*

☐ Family members did not agree *akumtundu sanagwirizane nazo*

☐ Too many referrals *kunachuluka kopita kuti tikalandire thandizo*

☐ Cannot take time from work/duties *nthawi ndinalibe*

☐ I forgot *ndinaiwala*

☐ Afraid *ndimachita mantha*

☐ Other (please specify) *zifukwa zina (Tchulani)*

If transport difficulties, was it? (check all that apply): *munali ndi vuto la mayendedwe?*

☐ No transport available *ma transport panalibe, amasowa*

☐ Can not physically carry child: *too heavy or difficult sindikanatha kumunyamula mwana, kulemera kapena kuvuta*

☐ Too far *kutali.*

☐ Not safe *sikwabwino*

☐ No one available to accompany the child *palnalibe opita naye mwana*

☐ Other (please specify) *zifukwa zina (Tchulani)*
